# Supplementary material for: Revisiting Schistosoma mansoni Micro-Exon Gene (MEG) Protein Family: A Tour into Conserved Motifs and Annotation
Source: Biomolecules. 2023 Aug 22;13(9):1275. doi: 10.3390/biom13091275 (PMC10526429; doi:10.3390/biom13091275)
Supplement: Supplementary file 1 [file biomolecules-13-01275-s001.zip › biomolecules-2542735-supplementary.pdf]

“Revisiting annotation of *Schistosoma mansoni* Micro-Exon Gene (MEG) family”

Štěpánka Nedvědová [1,2,3], Davide De Stefano [1], Olivier Walker [1], Maggy Hologne [1], Adriana Erica Miele [1,4*]

[1] UMR 5280 Institute of Analytical Sciences, Université de Lyon, CNRS, Université Claude Bernard Lyon 1, Villeurbanne, France
[2] Department of Chemistry, Faculty of Agrobiology, Food and Natural Resources, Czech University of Life Sciences, Prague, Czech Republic
[3] Department of Zoology and Fisheries, Center of Infectious Animal Diseases, Czech University of Life Sciences, Prague, Czech Republic
[4] Department of Biochemical Sciences, Sapienza University of Rome, Rome, Italy

*corresponding author. eMail: adriana.miele@univ-lyon1.fr

Supplementary Material

Supplementary Table S1: Summary of sequences and primary structure characteristics of MEG proteins analysed in this study.
Supplementary Figure S1: Multiple alignment of MEG proteins by MUSCLE

Supplementary Table S1: Summary of sequences and primary structure characteristics of MEG proteins analysed in this study. Parameters such as molecular weight (MW), aliphatic index and gravy index have been calculated by ProtParam on the EXPASY server.

**Chromosome number**	**Gene identifier and name in WBPS**	**GenBank ID / GenBank protein seq.**	**UniProt ID**	**MW (g/mol)**	**pI**	**Aliphatic index**	**Gravy Index**	**Protein Sequence**
1	*Smp_085840.1 MEG-4 Antigen 10.3*	8347941	C4QKE8	12908	4.86	89.16	-0.45	MNFLTLYVTLVYTILSVYSDIEPRIQKEYYYNLHENNSQANHNKFHEMPEYDDQLPDFPHKQLEEEQNPFHKLSEVLNSGSVVPLWLVNPIYYVLELFPRAISYYFN
1	*Smp_124000.1 MEG-14 isoform 3*	AOL57991.1	A0A1C9A1H6	14354	10.31	80.5	0.1	MNRFFWTVTQCTILLVIICNLNTMKATSANSRTHGATSTSTHGATSTAKPAASTPPKAAATSTIKPTVTTPKAAATSTIKPTVTTSKPSPAKPAASNTAKPAASTPKKPHDERAVLAAAAVPIVLGVIGEVIGFILQYIAS
1	*Smp_124000.1 MEG-14 isoform 6*	AOL57994.1	A0A1C9A1I5	13820	10.19	76.99	0.05	MNRFFWTVTQCTILLVIICNLNTMKATSANSRTHGATSTTHGATSTAKPAASTPPKAAATSTIKPTVTTPKAAATSTTEPTVTTSKPSPAKPAASNTAKPAASTPKKPHDERAVLAAAAVPIVLGVIGEVIGFILQ
1	*Smp_124000.1 MEG-14 isoform 7*	AOL57995.1	A0A1C9A1J0	14760	10.56	74.72	-0.06	MNRFFWTVTQRTILLVIICNLNTMKATSANSRTHGATSTRTHGATSTAKPAASTPIKPTVTTPKAAATSTTEPTVTTPKAAATSTTEPTVTTSKPSPAKPAASNTAKPAASTPKKPHDERAVLAAAAVPIVLGVIGEVIGFILQ
1	*Smp_124000.2 MEG-14*	AAN17279.1	Q8ITD5	15822	10.4	79.04	0.09	MNRFFWTVTQCTILLVIICNLNTMKATSANSRTHGATSTSTHGATSTAKPAASTPPKAAATSTIKPTVTTPKAAATSTIKPTVTTPKAAATSTIKPTVTTSKPSPAKPAASNTAKPAASTPKKPHDERAVLAAAAVPIVLGVIGEVIGFILQYIAS
1	*Smp_124000.2 MEG-14 isoform 1*	AOL57989.1	A0A1C9A1I1	14267	10.31	81.07	0.11	MNRFFWTVTQCTILLVIICNLNTMKATSANSRTHGATSTSTHGATSTAKPAASTPPKAAATSTIKPTVTTPKAAATSTIKPTVTTKPSPAKPAASNTAKPAASTPKKPHDERAVLAAAAVPIVLGVIGEVIGFILQYIAS

1	*Smp_124000.2 MEG-14 isoform 2*	AOL57990.1	A0A1C9A1I4	15782	10.16	74.52	0.01	MNRFFWTVTQCTILLVIICNLNTMKATSANSRTHGATSTRTHGATSTAKP AASTPPKAAATSTIKPTVTTPKAAATSTTEPTVTTPKAAATSTTEPTVT-TKPSPAKPAA SNTAKPAASTPKKPHDERAVLAAAAVPIVLGVIGEVIGFILQYIAS
1	*Smp_124000.2 MEG-14 isoform 3*	N/A	A0A5K4EK08	15509	10.03	83.47	0.1	YLLCKKEYSIMNRFFWTVTQCTILLVIICNLNTMKATSANSRTHGATSTSTHGATSTAKP AASTPPKAAATSTIKPTVTTPKAAATSTIKPTVTTKPSPAKPAASNTAKPAASTPKKPHDERAVLAAAAVPIVLGVIGEVIGFILQYIAS
1	*Smp_124000.2 MEG-14 isoform 4*	AOL57992.1	A0A1C9A1I3	15447	10.25	75.33	0.01	MNRFFWTVIQCTILLVIICNLNTMKATSANSRTHGATSTRTHGATSTAKP AASTPPKAAATSTIKPTVTTPKAAATSTTEPTVTTPKAAATSTTEPTVTTSKPSPAKPAA SNTAKPAASTPKKPHDERAVLAAAAVPIVLGVIGEVIGFILQ
1	*Smp_124000.2 MEG-14 isoform 5*	AOL57993.1	A0A1C9A1I0	14179	10.31	81.65	0.12	MNRFFWTVTQCTILLVIICNLNTMKATSANSRTHGATSTTHGATSTAKPAASTPPKAAATSTIKPTVTTPKAAATSTIKPTVTTKPSPAKPAASNTAKPAASTPKKPHDERAVLAAAAVPIVLGVIGEVIGFILQYIAS
1	*Smp_165050.1 MEG-7*	8345678	G4V7W5	13106	10.04	79.74	-0.36	MNTLFRSIFVVVVVVYAYFDMANGVPEPPRVPDEDAVPVRAKPATPIKISTDKIPVNKTMKIQTTPSKEKKQKPDPKRYKRSSYQKDKKAKSSSSTLTIGYPILFITTPFVISKFLL
1	*Smp_165050.2 MEG-7 iso2*	N/A	A0A5K4EUJ7	16568	9.88	82.62	-0.43	MSVPIRNLLRNNEYAICKFQYCKHIDNGNYNILQRSIFVVVVVVYAYFDMANGVPEPPRVPDEDAVPVRAKPATPIKISTDKIPVNKTMKIQTTPSKEKKQKPDPKRYKRSSYQKDKKAKSSSSTLTIGYPILFITTPFVISKFLL
1	*Smp_176020.1 MEG-11*	N/A	A0A3Q0KTG4	8635	5.36	81.52	0.03	MKLTHILLICFISFLFFTYVQCDGEEEENEEEEKPPQPDVPHGKHPLLRKAFLTAPSWLHMPFSIAGAVAAYVFYHFYG

1	*Smp_243780.1 MEG-30*	CUS27856.1	A0A0U5KI45	6687	8.14	119.31	0.66	MQVDKFIIYTIVIIAIAIFVSMPEIHAFGIKFFTTTPVPNKGLLDKLLDGLYQFFNRH
1	*Smp_243790.1 MEG-31 protein*	CUS27857.1	A0A0U5KFM1	6879	9.39	88.1	-0.21	MHCVLLLLSLFAVCSVIMPTVKSGGSASGGSTEVDLMHKRGKDREDKRRKDYIKELVKNATGT
1	*Smp_307220.1 MEG-4 Antigen 10.3*	N/A	A0A5K4F627	19788	6.73	83.35	-0.64	MNIYLIGILCIVGLIISQGSTANGSPLDDRFNDVNTINKKQFTEEEFSRLINSMLKEYIEDNKKDKHPTQKTTPKPTTPKQINDGTSDKTSDTHTIKRTTPKPTTPKQINDGTSDKPKSIADIFLINKPKVPLWIVNPLYYMVEKFVQIMGYLLEDDDTLELNLPKYYYDKSI
1	*Smp_307220.2 MEG-4 Antigen 10.3*	AAP13803.1	Q86D79	22739	8.43	76	-0.77	MNIYLIGILCIVGLIISQGSTANGSPLDDRFNDVNTINKKQFTEEEFSRLINSMLKEYIEDNKKDKHPTQKTTPKPTTPKQINDGTSDKTSDTHTIKRTTPKPTTPKQINDGTSDKTSDTHTIKRTTPKPTTPKQINDGTSDKPKSIADIFLINKPKVPLWIVNPLYYMVEKFVQIMGYLLEDDDTLELNLPKYYYDKSI
1	*Smp_307220.3 MEG-4 Antigen 10.3*	N/A	A0A5K4F2K5	20828	7.74	81.37	-0.67	MNIYLIGILCIVGLIISQGSTANGSPLDDRFNDVNTINKKQFTEEEFSRLINSMLKEYIEDNKKDKHPTQKTSDTHTIKRTTPKPTTPKQINDGTSDKTSDTHTIKRTTPKPTTPKQINDGTSDKPKSIADIFLINKPKVPLWIVNPLYYMVEKFVQIMGYLLEDDDTLELNLPKYYYDKSI
1	*Smp_307240.1 MEG-4 Antigen 10.3*	N/A	A0A5K4F4B1	22129	9.14	94.17	-0.59	MKLVSISLIGIFSLLISQEYGYLIDIKHINSPNQKQYVRDKMNLLNEYLTSRNIKKQFTEEEFSRLINSMLKKHIEDKNVDIRIIENKKDKHPTQKTSDTHTIKRTTPKPTTPKQINDGTSDKPKSIADFFLINKPKVPLWIVNPLYYMVEKFVQIMGYLLEDDDTLELNLPKYYYDKSI
2	*Smp_171190.1 MEG-8*	8355812	G4VCW5	20959	9.86	66.86	-0.55	MFTIILIYVLYFIANAKFEHTTSGIRNPSKLSDSNASKTLSLKNLTDHYIHTPQKSNNGTSCNGKDTCKLPNPSQKGFTNTTSLPHTQSHNSTVAPSVPKPTRQEIPRSGTIVNGTKPTPGKPVVNGTKPTPGKPESFLKRVGDGFFDLFSEQEFHPINHKSYLFNFWYLFRTSFLNLKNMKNLLLGS

3	*MEG-2.1 iso1*	ADI31109.1	D7DP78	9792	5.53	86.36	-0.05	MKLSGANCLVVFSLLQLLVAFSHCDINDITCNKTVCCASEDGKKGSLCCEKDGCPIPSTPDLLLGNYQRHQRMKNYLEEVCENFIYTP
3	*MEG-2.1 iso2*	ADI31107.1	D7DP76	5641	4.83	103.08	0.51	MKLSGANCLVVFSLLQLLVAFSHCDINDITCNKTVCCASEDGKIGENFIYTP
3	*MEG-2.1 iso3*	ADH02222.1	D7PD75	2818	7.98	146.15	1.14	MKLSGANCLVVFSLLQLLVALSHYTP
3	*Smp_122630.1 MEG-1 iso1*	N/A	A0A3Q0KKC4	18213	5.34	88.65	-0.09	MANKDLILTPYQVFILPCFILIFWSLFLIVFKSDGSGTWRKDIDWLILTKGGKLNRTWFVFNETKEICSCLTDFIKCIFREINIDKDYLCTYPTNFSHGLITYCTKSNDERDLLSYEEDHIALYVIQPTNHCQRYEGSSSLVSQKPEKECPFCFD
3	*Smp_122630.1 MEG-1 iso10*	ADH02235.1	D7PD88	17319	5.58	87.57	-0.07	MANRDLILTPYQVFILPCFILIFWSLFLIVFKSDGSGTWRKDIDWLILTKGGKLNRTWFVFNETKEICSCLTDSIKCIFREICTYPTNFSHGLITYCTKSNDERDLLSYEEDHIALYVIQPTNHCQRYEGSSSLVSQKPEKECPFCFD
3	*Smp_122630.1 MEG-1 iso11*	ADH02236.1	D7PD89	16526	5.58	89.15	-0.07	MANRDLILTPYQVFILPCFILIFWSLFLIVFKSDGSGTWRKDIDWLILTKGGKLNRTWFVFNETKEICSCLTDFIKCIFREISHGLITYCTKSNDERDLLSYEEDHIALYVIQPTNHCQRYEGSSSSVSQKPEKECPFCFD
3	*Smp_122630.1 MEG-1 iso16*	ADH02240.1	D7PD93	17523	5.33	86.98	-0.12	MANRDLILTPYQVFILPCFILIFWSLFLIVFKSDGSGTWRKDIDWLILTKGGKLNRTWFVFNETKEICSCCIFREINIDKDYLCTYPTNFSHGLITYCTKSNDERDLLSYEEDHIALYVIQPTNHCQRYEGSSSLVSQKPEKECPFCFD
3	*Smp_122630.1 MEG-1 iso18*	ADH02241.1	D7PD94	14425	5.28	98.28	0.06	MANRDLILTPYQVFILPCFILIFWSLFLIVFKSDGSGTWRKDIDWLILTKGGKLNRTWFVFNETKEICSCCIFREINIDKDYLCTYPTNFSHGLITYCTKSNDERDLLSYEEDHIALYVIQPTNHCQRYEGSSSLVSQKPEKECPFCFD
3	*Smp_122630.1 MEG-1 iso5*	ADH02230.1	D7PD83	18241	5.34	88.65	-0.09	MANRDLILTPYQVFILPCFILIFWSLFLIVFKSDGSGTWRKDIDWLILTKGGKLNRTWFVFNETKEICSCLTDFIKCIFREINIDKDYLCTYPTNFSHGLITYCTKSNDERDLLSYEED

								HIALYVIQPTNHCQRYEGSSSLVSQKPEKECPFCFD
3	*Smp_122630.1 MEG-1 iso6*	ADH02231.1	D7PD84	16143	5.48	91.88	-0.1	MANRDLILTPYQVFILPCFILIFWSLFLIVFKSDGSGTWRKDIDWLILTKGGKLTDFIKCIFREINIDKDHLCTYPTNFSHGLITYCTKSNDERDLLSYEEDHIALYVIQPTNHCQRYEGSSSLVSQKPEKECPFCFD
3	*Smp_122630.1 MEG-1 iso8*	ADH02233.1	D7PD86	14480	5.53	95.89	-0.02	MANRDLILTPYQVFILPCFILIFWSLFLIVFKSDGSGTWRKDIDWLILTKGGKLTDFIKCIFREISHGLITYCTKSNDERDLLSYEEDHIALYVIQPTNHCQRYEGSSSLVSQKPEKECPFCFD
3	*Smp_122630.2 MEG-1 iso2*	N/A	A0A5K4EKN1	18156	6.28	102.53	0.02	MANKDLILTPYQVFILPCFILIFWSLFLIVFKSDGSGTWRKDIDWLILTKGGKLNRTWFVFNETKEICSCLTDFIKCIFREINIDKDYLCTYPTNFSHGLITYCTKSNDERDLLSYEEDHIALYVIQPTNHCQRYEGSSIKKRLLESYLIITPI
3	*Smp_122630.2 MEG-1 isoform 1*	ADH02226.1	D7PD79	18184	6.28	102.53	0.02	MANRDLILTPYQVFILPCFILIFWSLFLIVFKSDGSGTWRKDIDWLILTKGGKLNRTWFVFNETKEICSCLTDFIKCIFREINIDKDYLCTYPTNFSHGLITYCTKSNDERDLLSYEEDHIALYVIQPTNHCQRYEGSSIKKRLLESYLIITPI
3	*Smp_122630.2 MEG-1 isoform 12*	ADH02243.1	D7PD99	16495	6.81	107.21	0.09	MANRDLILTPYQVFILPCFILIFWSLFLIVFKSDGSGTWRKDIDWLILTKGGKLNRTWFVFNETKEICSCLTDFIKCIFREISHGLITYCTKSNDERDLLSYEEDHIALYVIQPTNHCQRYEGSSIKKRLLESYLIITPI
3	*Smp_122630.2 MEG-1 isoform 14*	ADH02238.1	D7PD91	16112	6.27	107.45	0.04	MANRDLILTPYQVFILPCFILIFWSLFLIVFKSDGSGTWRKDIDWLILTKGGKLTDFIKCIFREINIDKDYLCTYPTNFSHGLITYCTKSNDERDLLSYEEDHIALYVIQPTNHCQRYEGSSIKKRLLESYLIITPI
3	*Smp_122630.2 MEG-1 isoform 17*	ADH02242.1	D7PD95	15394	6.27	106.41	0.02	MANRDLILTPYQVFILPCFILIFWSLFLIVFKSDGSGTWRKDIDWLILTKGGKCIFREINIDKDYLCTYPTNFSHGLITYCTKSNDERDLLSYEEDHIALYVIQPTNHCQRYEGSSIKKRLLESYLIITPI
3	*Smp_138060.1*	N/A	A0A3Q0K	16404	4.81	57.55	-0.32	MLFFALILIISLHSFDCAFTAQQECEKNCKGDNEYVSPNC

	MEG-3 Grail family		MS0					GILCSGTIGPQTFYCYLGCSHNATKQSEFDNCKTKCDGGVQLTKEACLSNCGLITTHPELCDAVCGGNDGGSFPICLYNCDQEHTDPRKDGADGSEDFDKCKTKCYKMAGQ
3	*Smp_138060.1 MEG-3.3 isoform 1*	ADH02209.1	D7PD62	16403	5.07	57.55	-0.32	MLFFALILIISLHSFDCAFTAQQECEKNCKGDNEYVSPNCGILCSGTIGPQTFYCYLGCSHNATKQSEFDNCKTKCDGGVQLTKEACLSNCGLITTHPELCDAVCGGNDGGSFPICLYNCDQKHTDPRKDGADGSEDFDKCKTKCYKMAGQ
3	*Smp_138060.1 MEG-3.3 isoform 2*	ADH02210.1	D7PD63	16153	4.81	55.78	-0.37	MLFFALILIISLHSFDCAFTAQQECEKNCKGDNEYVSPNCGILCSGTIGPQTFYYHYFSTKQSEFDNCKTKCDGGVQLTKEACLSNCGLITTHPELCDAVCGGNDGGSFPICLYNCDQEHTDPRKDGADGSEDFDKCKTKCYKMAGQ
3	*Smp_138060.1 MEG-3.3 isoform 3*	ADH02211.1	D7PD64	15542	4.7	57.34	-0.35	MLFFALILIISLHSFDCAFTAQQECEKNCKGDNEYVSPNCGILCSGTIGPQTFYSTKQSEFDNCKTKCDGGVQLTKEACLSNCGLITTHPELCDAVCGGNDGGSFPICLYNCDQEHTDPRKDGADGSEDFDKCKTKCYKMAGQ
3	*Smp_138070.1 MEG-3.2 (Grail) isoform2*	ADH02200.1	D7PD53	14811	5.6	60.72	-0.17	MLFVALILIISLHSFDCVFTARETQQECVRHCGGHSGGLCSGSTGPQTFYCYLGCSHNASNQNDFDKCLPKCNGSPQLTESSCQNDCGRVTTHPELCGIVCGGNVGDSFPLCLYNCDQGNGSGNFDECKTKCYLMAGR
3	*Smp_138070.1 MEG-3.2 (Grail) isoform2/1*	GB PS : ADH02199.1	D7PD52	15769	5.39	57.1	-0.26	MLFVALILIISLHSFDCVFTARETQQECVRHCGGHNEYVTRYCGGLCSGSTGPQTFYCYLGCSHNASNQNDFDKCLPKCNGSPQLTESSCQNDCGRVTTHPELCGIVCGGNVGDSFPLCLYNCDQGNGSGNFDECKTKCYEMAGR
3	*Smp_138070.1 MEG-3.2 (Grail) isoform3*	GB PS : ADH02201.1	D7PD54	15208	5.39	57.17	-0.3	MLFVALILIISLHSFDCVFTARETQQECVRHCGGHNEYVTRYCTGPQTFYCYLGCSHNASNQNDFDKCLPKCNGSPQLTESSCQNDCGRVTTHPELCGIVCGGNVGDSFPLCLYNCDQGNGSGNFDECKTKCYEMAGR
3	*Smp_138070.1 MEG-3.2 (Grail) isoform6*	ADH02204.1	D7PD57	14794	5.62	55.88	-0.27	MLFVALILIISLHSFDCVFTARETQQECVRHCGGHNEYVTRYCGGLCSGSTGPQTFYCYLGCSHNASNQNDFDKCLPKCNGQNDCGRVTTHPELCGIVCGGNDGGSFPICLYNCDQGNGSGNFDECKTKCYEMAGR

3	*Smp_138070.1 MEG-3.2 (Grail) isoform9*	ADH02207.1	D7PD60	14500	5.42	54.62	-0.34	MLFVALILIISLHSFDCVFTARETQQECVRHCGGHNEYVTRYCGGLCSGSTGPQTFYCYLGCSHNASNQNDFDKCLPKCNGQNDCGRVTTHPELCGIVCGGNDGGSFPICLYNCDQGNGSGNFDECKTKCYEMAGR
3	*Smp_138070.2 MEG-3.2 (Grail) isoform1*	N/A	A0A5K4EPC8	17562	7.16	68.01	-0.22	RTTTHRLVKMLFVALILIISLHSFDCVFTARETQQECVRHCGGHNEYVTRYCGGLCSGSTGPQTFYCYLGCSHNASNQNDFDKCLPKCNGSPQLTESSCQNDCGRPHTLNCVVSFVVEMLETHFHCVCITAIREMVRETLTNVKQSATKWRDGEFP
3	*Smp_138080.1 MEG-3 (Grail)*	N/A	A0A3Q0KMU6	17152	5.44	50.99	-0.67	MLFVALILIISLHSFDCVFTAQETRDAERECKKHCEGNNEYVTRYCGGLCSSNTGPQTFYCYLGCSHNASTQDDFDKCLPKCNDRVQLTEENCRDDCGRVTSHHELCGDVCGGNHGGSFPLCLYNCDQEHPREYERGYDKCKTKCYAMEGR
3	*Smp_138080.1 MEG-3.1 (Grail) isoform1*	ADH02196.1	D7PD49	16404	5.07	57.55	-0.32	MLFVALILIISLHSFDCVFTAQETRDAERECKKHCEGNNEYVTRYCGGLCSSNTGPQTFYCYLGCSHNASTQDDFDKCLPKCNDRVQLTEENCRDDCGRVTSHHELCGDVCGGNHGGSFPLCLYNCDQEHPREYERGYDKCKTKCYAMEGR
3	*Smp_138080.1 MEG-3.1 (Grail) isoform2*	ADH02197.1	D7PD50	14569	5.39	55.04	-0.44	MLFVALILIISLHSFDCVFTAQETRDAERECKKHCEGNNEYVTRYCGGLCSSNTGPQTFYCYLGCSHNASTQDDFDKCLPKCNDRVQLTEENCRNDCGRVTSHHELCGDVCGGNHGGSFPLCFFQSSSSDK
3	*Smp_138080.1 MEG-3.1 (Grail) isoform3*	ADH02198.1	D7PD51	16615	5.77	47.07	-0.74	MLFVALILIISLHSFDCVFTAQETRDAERECKKHCEGNNEYVTRYCGGLCSSNTGPQTFYCYLGCSHNASTQDDFDKCLPKCNDRVQLTEENCRNDCGRVTSHHESCGDVCGGNHGGSFPLCSYNCDQEHPREYERGKTKRYAMEGR
3	*Smp_159800.1 MEG-2 (ESP15) family*	8347467	C4QG05	9095	7.53	95.06	0.03	MCLTIFYVIHLLAIFSDSTEWVITCNKTTCCDEDGNSKICCVGNDCKDVIKPRSSGADDLNLFLRKRGMAYKLGEILKKLN
3	*Smp_159800.1 MEG-2.4*	ADH02216.1	D7PD69	8971	6.52	96.3	0.05	MCLTIFYVIHLLAIFSDSTEWVITCNKTTCCDEDGNSKICCVGNDCKDVIKPRSSGADDLNLFLRKRGMAYKLGEILK

	isoform 1							KLN
3	*Smp_159830.1 MEG-2 (ESP15) family*	N/A	A0A3Q0KR24	7734	5.07	87.54	-0.05	MCLTIFYVIHLLAIFSDSNEWVITCNKTTCCDEDKNSKICCVGNDCKDVIKPRSSGADDFDLLKKLNSP
3	*Smp_180310.1 MEG-2 (ESP15)*	8340871	C4QPR6	8646	7.54	50.68	-0.58	MERFKSSYFYFEIYLLCFTETVCCESEDGKAGSLCCEKNGCSVPSGTHDLLSENYRRHQRMKNYLKEVCKYFK
3	*Smp_180320.1 MEG-2 (ESP15 iso1)*	8340874	C4QPR8	11165	7.57	73.02	-0.22	MHGIWCKVPVSVVIWIHSTLFQFTFKVFYELKQNNTFPLPGDGWTITCNETYCCENTDNGKLCCDGEYCSASISKLDPPFSNCFQYVFVS
3	*Smp_180320.2 MEG-2 (ESP15 iso2)*	N/A	A0A3Q0KTV3	10983	6.87	73.79	-0.2	MHGIWCKVPVSVVIWIHSTLFQFTFKVFYELKQNNTFPLPGDGWTITCNETYCCENTDNGKLCCDGEYCSASISNHQDLTKHQQNLLMSKKFKII
3	*Smp_180330.1 MEG-2 (ESP15 family)*	8340875	C4QPR9	5497	10.37	103.54	-0.35	QKAISQRPFAVIKMVVTVDNPELTLKNYLRKAQMIDKLREAVQKLGGR
3	*Smp_180340.1 MEG-2 (ESP15)*	8340876	C4QPS0	4895	9.9	84.09	0.14	MTAKGSVAMASFVLVYDPSVAVKNYRQQVLMATKIKEVCQKFRG
3	*Smp_326790.1 MEG-1*	N/A	A0A5K4F8B3	22040	5.12	79.09	-0.3	MAKSDLILTPYQVFILPCIILIFWSLFLIVFKSDGSGTWRRDIDWFILTQGKQIQRIWFVFNETEEVCSCVTGFEKCNIREIIAANYSPIIHNEEKKNISNREKDYLCTCPTYFNHGVITYCTKSNYEKDSLQYEDDFMDLFVKKHNKDDCQHYGGYSSLEYRNPEKVCPFCYEEITQCSIWETLS
3	*Smp_326790.2 MEG-1*	N/A	A0A5K4F8U8	21198	5.24	80.56	-0.34	MAKSDLILTPYQVFILPCIILIFWSLFLIVFKSDGSGTWRRDIDWFILTQGKQIQRIWFVFNETEEVCSCVTGFEKCNIREIIAANYSPIIHNEEKKNISNREKDYLCTCPTYFNHGVITYCTKSNYEKDSLQYEDDFMDLFVKKHNKDDCQHYGGY

								SSLEYRNPEKEITQCSIWETLS
3	*Smp_326790.3 Uncharacterized*	N/A	A0A5K4FAB4	19957	5.23	65.33	-0.67	MYYPYFLVSIFDSLQNQSYIKQGSGTWRRDIDWFILTQGKQIQRIWFVFNETEEVCSCVTGFEKCNIREIIAANYSPIIHNEEKKNISNREKDYLCTCPTYFNHGVITYCTKSNYEKDSLQYEDDFMDLFVKKHNKDDCQHYGGYSSLEYRNPEKEITQCSIWETLS
3	*Smp_336990.1 Uncharacterized*	N/A	A0A5K4FDB9	9393	6.68	82.17	-0.07	MKLSGANCLVVFSLLQLLVAFSHCDISDITCNKTVCCASEDGKTGSLCCEKDGCPSTPDLFLENYRRHRRMKNYLEEVCKYYI
3	*Smp_345100.1 MEG-2.2 isoform1*	N/A	A0A5K4FFX0	13413	9.64	96.52	-0.37	MYCQSFTLLNRDYISNVTKQSKHRLYNTMKLSGANCLVVFSLLQLLVAFSHCKLMSHNMQQDSLLRQKTVKKVRTEERWLSNTPDLLLGNYQRHQRMKNYLEEVQILHIYYI
3	*Smp_345100.1 MEG-2.2 isoform2*	ADH02224.1	D7PD77	9336	7.5	87.37	0.02	MKLSGATCLVVFSLLQLLVAFSHCDISAITCNKTVCCASEDGKTGSLCCEKDGCPSTPDLFLENYRRHRRMKNYLEEVCKYYI
4	*Smp_158890.1 MEG-16 iso1*	N/A	A0A3Q0KQX7	12759	10.12	78.68	-0.18	MFYCRVLIITSFMIFLLGTANCDIIDVLSLLFGGNGNKNIRRNRNRGGDSGGLSDFLTSLFDWNGDGYRGSGFNFYDFLSLFFGLNKKDNRNNRRYRSGGGGGNGGLIRLFFAR
4	*Smp_158890.2 MEG-16 iso2*	N/A	A0A5K4EU45	11802	9.91	72.21	-0.32	MFYCRVLIITSFMIFLLGTANCDIIDDKNIRRNRNRGGDSGGLSDFLTSLFDWNGDGYRGSGFNFYDFLSLFFGLNKKDNRNNRRYRSGGGGGNGGLIRLFFAR
5	*Smp_152580.1 MEG-5*	8345024	G4LYD1	8884	9.36	112.28	0.05	MRRNYLLLYICIIVFILLKEINASGRQPKFVNVDTDGNLRSGGSSDISDMFGQNKTLGTAFKTLLHNLWDLLKQSLGLP
5	*Smp_152590.1 MEG-10*	N/A	A0A3Q0KQ39	6127	7.74	59.29	0.04	MTLLLIQSCHCGSSGSTEAGSNGTNSKGWWPKFLGWANTFCTFITFSNTIQNFIYG
5	*Smp_152590.2 MEG-10 iso2*	8345025	G4LYD0	6056	7.74	58.55	0.01	MTLLLIQSCHCGSSGSTEGSNGTNSKGWWPKFLGWANTFCTFITFSNTIQNFIYG
5	*Smp_152630.1 MEG-12*	N/A	A0A3Q0KQ41	5050	8.1	60.24	-0.18	GENYEQQLQQPKAYGIWSLFSYFYKTFKVFCSVSNMVNWIFG

5	*Smp_243730.1 MEG-10.2 protein*	CUS27851.1	A0A0U5KJN7	7428	5.96	88.44	0.36	MISLLLFGLLLLQSCLYCSSDNENAGTTTEKPTSFWKRFFDFFNFICTLNQTWSTIRNFFGIAL
5	*Smp_243750.1 MEG-27 iso1*	CUS27853.1	A0A0U5KIV9	6701	5.99	95.64	0.26	MNLIQTLLWMIFMMIMNLTNEIKWVNCSHELNEHTSETSLRGWIHTVFSFLFHNF
5	*Smp_243750.1 MEG-27 iso2*	N/A	A0A5K4F014	6800	6.38	95.64	0.18	MNLIQTLLWMIFMMIMNLTNEIKWVNCSHELNEHTSETSLRRWIHTVFSFLFHNF
6	*Smp_123100.1 MEG-32.1*	CUS27858.1	A0A0U5KEW2	8861	9.3	118.72	0.61	MYYRHYLLAIINVIVLSTMIQYVIGGSIFGDDTSTTKNMTTTTKASSANSLEVSWLAISSISMIVIGLINGHLRRFIF
6	*Smp_123200.1 MEG-32.2*	CUS27859.1	A0A0U5KJ28	9879	6.63	102.81	0.26	MKETTVMHHYHHPHPNHRLLTVISAIVLLTIVHDVKGSGLFDDDITTRTTAPTTTSGSVSSFQVSWLALSSVFMIVLGLINSYTERSIF
6	*Smp_127990.1 MEG 13 iso1*	N/A	A0A3Q0KLA7	14352	4.19	58.46	-0.41	MDITYSWCIICLINLLLNGKLGQAQEDNYTEDSTTDPTTFDNTTVTSTTTEFNNTTVTSTTTEFNNTTVTSTTTEFTNKPKVENSTTDGTTYTTTPSHFSTSTSTNDATNSKFQRIFYMIVGLISLMAIN
6	*Smp_127990.2 MEG 13 iso2*	N/A	A0A5K4EL02	13790	4.27	60.8	-0.39	MDITYSWCIICLINLLLNGKLGQAQEDNYTEDSTTDNTTVTSTTTEFNNTTVTSTTTEFNNTTVTSTTTEFTNKPKVENSTTDGTTYTTTPSHFSTSTSTNDATNSKFQRIFYMIVGLISLMAIN
6	*Smp_172180.1 MEG-8*	8340626	G4VLP3	15839	9.07	58.5	-0.47	MNTVTLGLFCIAICLIGINAGTVSKPTATVKPQPVNKMNTTPVHQEEPSFWRRMWNSFTSMFGSSDSSSGTNNKDTKSPNPNTTEAKSLSLKERIMNKFNSIFGEEEYNPPKDSDFTERLWMLFKHCFLNFKNLAKIFST
6	*Smp_243740.1 MEG-26 protein*	CUS27852.1	A0A0U5FZ31	8252	7.75	129.45	0.25	MDISKILLGSLFLLSVIILQEVNGQKGNRVIFNVEELILNLWKNLYERLADTFKCLLSPLPESIGGKNKSCYP
6	*Smp_243760.1 MEG-28*	CUS27854.1	A0A0U5KKP6	7864	10.01	110.76	0.44	MNTIVRYYLIILFIITTIEIQNIRSAFKKRPPASFVILENMTSTDRFRKLLYHCFTSFSTWMVLLG
6	*Smp_243770.1*	CUS27855.	A0A0U5K	7636	6.7	129.86	0.94	MLNKLLLQLFILVTIIIIHDVKCGGEEETTTTTLPTTTSVAI

	MEG-29 protein	1	LL2					KGTISAYTVMMGLSIYVIHSFIVFKMM
7	*Smp_010550.1 Uncharacterized MEG-15 iso4*	N/A	A0A3Q0KC91	16111	9.94	86.31	-0.44	MLNRFIVILVFVFVGIVTFDNVQGQRDPPRTNNTITHTTNHYVGKLSHHNTVPAKTTRKSQHPNTTPSHTDKTVQKKCLNKMTPQDLISLLFSLIPQIKTIEFSQNENLLKLATILEKIFEQQSRVEHSSPTKTPANKIFH
7	*Smp_010550.2 Uncharacterized MEG-15 iso2*	N/A	A0A5K4E9M7	18948	10.26	81.08	-0.52	MLNRFIVILVFVFVGIVTFDNVQGKTNNTITHTTNHYVGKLSHHNTVPAKTTRKSQHTTATARHHNTLKTTLSHHNTVPAKTTRKSQHPNTTPSHTDKTVQKKCLNKMTPQDLISLLFSLIPQIKTIEFSQNENLLKLATILEKIFEQQSRVEHSSPTKTPANKIFH
7	*Smp_010550.3 Uncharacterized MEG-15 iso1*	8353067	G4VMN2	19569	10.33	78.72	-0.59	MLNRFIVILVFVFVGIVTFDNVQGQRDPPRTNNTITHTTNHYVGKLSHHNTVPAKTTRKSQHTTATARHHNTLKTTLSHHNTVPAKTTRKSQHPNTTPSHTDKTVQKKCLNKMTPQDLISLLFSLIPQIKTIEFSQNENLLKLATILEKIFEQQSRVEHSSPTKTPANKIFH
7	*Smp_010550.4 Uncharacterized MEG-15 iso3*	N/A	A0A5K4E9G8	16045	9.94	87.73	-0.37	MLNRFIVILVFVFVGIVTFDNVQGQRDPPRTNNTITHTTNHYVGKLSHHNTVPAKTTRKSQHTTATASHTDKTVQKKCLNKMTPQDLISLLFSLIPQIKTIEFSQNENLLKLATILEKIFEQQSRVEHSSPTKTPANKIFH
7	*Smp_125320.1 MEG-9*	N/A	A0A3Q0KKW2	6818	5.31	86.61	0.36	MIISCQFITGFVVHESSTEGQNHEELAAAAGAHFLQFLNGCFLNMDNLKKLVFPG
ZW	*Smp_163710.1 MEG-6*	8355717	G4VTX1	7549	12.14	98.77	-0.66	MVQNPKNTKKINRTIRRSTKTVIVITDRVQNIVLGHRLLHHRIPTIKRSKSHGINKNETVSNLFP

Supplementary Figure S1. Multiple alignment of the 87 sequences of MEG products by MUSCLE.

```
                                     cov    pid   1 [        .         .         .         .         :         .         .         .         .         100
 1 A0A0U5KKP6_MEG-28              100.0% 100.0%     ------------MNTIVRYYLIILF --------------------------------------------------------------------------
 2 A0A0U5FZ31_MEG-26               51.5%   1.9%     ------------MD-ISKILLGSLFLL-------------SVIILQEVNGQKGNRVI-FNVEELI---LNL--------WKN-LYERLAD----------
 3 C4QKE8_MEG-4                    71.2%   2.5%     ------------MN-FLTLY-----VTLVYTI--------LSVYSDIEPRIQKEYYYN-LHENNSQANHNKFHEMPE-------------KKDKHP----
 4 A0A5K4F4B1_MEG-4               100.0%   5.6%     MKLVSISLIGIFSLLISQEYG--------YLI--DIKHINSPNQKQYVRDKMNLLNE-YLTSRNI-KKQFTEEEFSRLINSMLK---------KHIED--
 5 A0A5K4F2K5_MEG-4                98.5%   4.4%     ------------MNIYLIGIL-CIVGL---------------IISQGSTANGSPLDDRFNDVNTINKKQFTEEEFSRLINSMLKEYIEDNKKDKHPTQKT
 6 A0A5K4F627_MEG-4                98.5%   4.6%     ------------MNIYLIGIL-CIVGL---------------IISQGSTANGSPLDDRFNDVNTINKKQFTEEEFSRLINSMLKEYIEDN----------
 7 Q86D79_MEG-4                   100.0%   4.0%     ------------MNIYLIGIL-CIVGL---------------IISQGSTANGSPLDDRFNDVNTINKKQFTEEEFSRLINSMLKEYIEDNKKDKHPTQKT
 8 A0A3Q0KQX7_MEG-16               98.5%   1.7%     MFYCRVLIITSFMIFLLGTAN-C------------------DIIDVLSLLFGGNGNKNIRRN-----RNRGGDSGGLS---------------------
 9 A0A5K4EU45_MEG-16               92.4%   2.9%     -------------------MFYCRVLI----I---------TSFMIFLLGTANCDIIDDKNIRRN---RNRGGDSGGLS---------------------
10 A0A3Q0KLA7_MEG-13               93.9%   4.5%     -----------MDITYSW----CIICL----I---------NLLLNGKLGQAQEDNYTEDSTTDP---TTFDNTTVTSTTTE------------------
11 A0A5K4EL02_MEG-13              100.0%   5.6%     -----------MDITYSW----CIICL----I---------NLLLNGKLGQAQEDNYTEDSTTDN---TTVTSTTTE-----------------------
12 A0A3Q0KQ39_MEG-10               42.4%   6.9%     ----------MTL----LLIQSCHCGS----S---------GSTEAGSNGTNSKGWWPKFLGWAN-----------------------------------
13 G4LYD0_MEG-10                   40.9%   6.9%     ----------MTL----LLIQSCHCGS----S---------GSTE-GSNGTNSKGWWPKFLGWAN-----------------------------------
14 G4VCW5_MEG-8                    98.5%   2.6%     MFT---------------IIL-IYVLY----F---------IANAKFEHTTSGIRNPSKLSDSNA----SKTLSLKNLTDHYIHTPQKSNNGTSCNGKDT
15 A0A3Q0KKW2_MEG-9                69.7%   5.6%     ------------------MIISCQ-------F---------ITGFVV-----------------------------------------------------
16 G4VLP3_MEG-8                   100.0%   5.0%     -----------MN--TVTLGLFCIAIC----L---------IGINAGTVSKPTATVKPQPVNKMN---TTPVHQ-EEPSFWRRMWNSFTSMFGSSDSSS-
17 G4V7W5_MEG-7                   100.0%   5.2%     -----------MN----TL------------F---------RSIFVVVVVYAYFDMANGVPEPPRVPDEDAVP--VRA-------KPATP-----IK-I-
18 A0A5K4EUJ7_MEG-7-iso2           98.5%   2.7%     --MSVPIRNLLRNNEY---AICKFQYCKH--IDNGNYNILQRSIFVVVVVYAYFDMANGVPEPPRVPDEDAVP--VRA-------KPATP-----IK-I-
19 A0A5K4EKN1_MEG-1                95.5%   4.5%     ---MANKDLILTP--YQVFILPCFILI----F---------WSLFLIVFKSDGSGTWRKDIDWLI---LTKGGKLNRTWFVFNETKEICSCLTDFIKCI-
20 D7PD99_MEG-1                    95.5%   4.9%     ---MANRDLILTP--YQVFILPCFILI----F---------WSLFLIVFKSDGSGTWRKDIDWLI---LTKGGKLNRTWFVFNETKEICSCLTDFIKCI-
21 D7PD79_MEG-1                    95.5%   4.5%     ---MANRDLILTP--YQVFILPCFILI----F---------WSLFLIVFKSDGSGTWRKDIDWLI---LTKGGKLNRTWFVFNETKEICSCLTDFIKCI-
22 D7PD91_MEG-1                    95.5%   5.0%     ---MANRDLILTP--YQVFILPCFILI----F---------WSLFLIVFKSDGSGTWRKDIDWLI---LTKGGK-----------------LTDFIKCI-
23 D7PD95_MEG-1                    95.5%   5.2%     ---MANRDLILTP--YQVFILPCFILI----F---------WSLFLIVFKSDGSGTWRKDIDWLI---LTKGG-----------------------KCI-
24 D7PD89_MEG-1                    98.5%   4.9%     ---MANRDLILTP--YQVFILPCFILI----F---------WSLFLIVFKSDGSGTWRKDIDWLI---LTKGGKLNRTWFVFNETKEICSCLTDFIKCI-
25 A0A3Q0KKC4_MEG-1                98.5%   4.5%     ---MANKDLILTP--YQVFILPCFILI----F---------WSLFLIVFKSDGSGTWRKDIDWLI---LTKGGKLNRTWFVFNETKEICSCLTDFIKCI-
26 D7PD88_MEG-1                    98.5%   4.7%     ---MANRDLILTP--YQVFILPCFILI----F---------WSLFLIVFKSDGSGTWRKDIDWLI---LTKGGKLNRTWFVFNETKEICSCLTDSIKCI-
27 D7PD93_MEG-1                    98.5%   4.7%     ---MANRDLILTP--YQVFILPCFILI----F---------WSLFLIVFKSDGSGTWRKDIDWLI---LTKGGKLNRTWFVFNETKEICSC------CI-
28 D7PD94_MEG-1                    98.5%   4.7%     ---MANRDLILTP--YQVFILPCFILI----F---------WSLFLIVFKSDGSGTWRKDIDWLI---LTKGGKLNRTWFVFNETKEICSC------CI-
29 D7PD83_MEG-1                    98.5%   4.5%     ---MANRDLILTP--YQVFILPCFILI----F---------WSLFLIVFKSDGSGTWRKDIDWLI---LTKGGKLNRTWFVFNETKEICSCLTDFIKCI-
30 D7PD84_MEG-1                    98.5%   5.0%     ---MANRDLILTP--YQVFILPCFILI----F---------WSLFLIVFKSDGSGTWRKDIDWLI---LTKGGK-----------------LTDFIKCI-
31 D7PD86_MEG-1                    98.5%   5.6%     ---MANRDLILTP--YQVFILPCFILI----F---------WSLFLIVFKSDGSGTWRKDIDWLI---LTKGGK-----------------LTDFIKCI-
32 A0A5K4FAB4_Uncharacterized      95.5%   2.9%     ---M-----------YYPYFL---VSI----F---------DSLQNQSYIKQGSGTWRRDIDWFI---LTQGKQIQRIWFVFNETEEVCSCVTGFEKCN-
33 A0A5K4F8B3_MEG-1               100.0%   3.2%     ---MAKSDLILTP--YQVFILPCIILI----F---------WSLFLIVFKSDGSGTWRRDIDWFI---LTQGKQIQRIWFVFNETEEVCSCVTGFEKCN-
34 A0A5K4F8U8_MEG-1               100.0%   3.4%     ---MAKSDLILTP--YQVFILPCIILI----F---------WSLFLIVFKSDGSGTWRRDIDWFI---LTQGKQIQRIWFVFNETEEVCSCVTGFEKCN-
35 A0A0U5KI45_MEG-30               59.1%   8.6%     -----------MQ--VDKFIIYTIVII----A---------IAIFVSMPEIHAFGIK-------------------------------------------
36 A0A5K4E9G8_MEG-15               95.5%   2.8%     ---------MLNR--FIVILVFVFVGIVT--F---------DNVQGQRDPPRTNNTITHTTNHYV---GKLSHHNTVPAKTTRKSQH-------------
37 A0A5K4E9M7_MEG-15               93.9%   1.8%     ---------MLNR--FIVILVFVFVGI----------------VTFDNVQGKTNNTITHTTNHYV---GKLSHHNTVPAKTTRKSQHTTATARHHNTLK-
38 A0A3Q0KC91_MEG-15               95.5%   2.8%     ---------MLNR--FIVILVFVFVGIVT--F---------DNVQGQRDPPRTNNTITHTTNHYV---GKLSHHNTVPAKTTRKSQ--------------
39 G4VMN2_MEG-15                   95.5%   2.3%     ---------MLNR--FIVILVFVFVGIVT--F---------DNVQGQRDPPRTNNTITHTTNHYV---GKLSHHNTVPAKTTRKSQHTTATARHHNTLK-
40 A0Q3Q0KQ41_MEG-12               39.4%   2.4%     ----------GEN--YEQQLQQPKAYG----I---------WSLFSYFYKTFK-----------------------------------------------
41 A0A5K4EPC8_MEG-3.2             100.0%   1.9%     --RTTTHRLVKML--FVALILIISLHS----F---------DCVFTA---RETQQECVRHCGGHN---EYVTRYCGGLCSGSTGPQTFYCYLGCSHNAS-
42 D7PD63_MEG-3.3                 100.0%   4.8%     -----------ML--FFALILIISLHS----F---------DCAF------TAQQECEKNCKGDN---EYVSPNCGILCSGTIGPQTFYYH----YFST-
43 D7PD64_MEG-3.3                 100.0%   4.9%     -----------ML--FFALILIISLHS----F---------DCAF------TAQQECEKNCKGDN---EYVSPNCGILCSGTIGPQTFYS--------T-
44 D7PD62_MEG-3.3                 100.0%   4.6%     -----------ML--FFALILIISLHS----F---------DCAF------TAQQECEKNCKGDN---EYVSPNCGILCSGTIGPQTFYCYLGCSHNAT-
45 A0A3Q0KMS0_MEG-3               100.0%   4.6%     -----------ML--FFALILIISLHS----F---------DCAF------TAQQECEKNCKGDN---EYVSPNCGILCSGTIGPQTFYCYLGCSHNAT-
46 D7PD57_MEG-3.2                  86.4%   3.4%     -----------ML--FVALILIISLHS----F---------DCVFTA---RETQQECVRHCGGHN---EYVTRYCGGLCSGSTGPQTFYCYLGCSHNAS-
47 D7PD60_MEG-3.2                  86.4%   3.4%     -----------ML--FVALILIISLHS----F---------DCVFTA---RETQQECVRHCGGHN---EYVTRYCGGLCSGSTGPQTFYCYLGCSHNAS-
48 D7PD53_MEG-3.2                  86.4%   3.4%     -----------ML--FVALILIISLHS----F---------DCVFTA---RETQQECVRHC----------GGHSGGLCSGSTGPQTFYCYLGCSHNAS-
```

49	D7PD52_MEG-3.2	86.4%	3.2%	-----------ML--FVALILIISLHS----F---------DCVFTA---RETQQECVRHCGGHN---EYVTRYCGGLCSGSTGPQTFYCYLGCSHNAS-
50	D7PD54_MEG-3.2	86.4%	3.4%	-----------ML--FVALILIISLHS----F---------DCVFTA---RETQQECVRHCGGHN---EYVTRYC-------TGPQTFYCYLGCSHNAS-
51	D7PD51_MEG-3.1	84.8%	1.9%	-----------ML--FVALILIISLHS----F---------DCVFTAQETRDAERECKKHCEGNN---EYVTRYCGGLCSSNTGPQTFYCYLGCSHNAS-
52	A0A3Q0KMU6_MEG-3	90.9%	1.9%	-----------ML--FVALILIISLHS----F---------DCVFTAQETRDAERECKKHCEGNN---EYVTRYCGGLCSSNTGPQTFYCYLGCSHNAS-
53	D7PD49_MEG-3.1	90.9%	1.9%	-----------ML--FVALILIISLHS----F---------DCVFTAQETRDAERECKKHCEGNN---EYVTRYCGGLCSSNTGPQTFYCYLGCSHNAS-
54	D7PD50_MEG-3.1	68.2%	2.3%	-----------ML--FVALILIISLHS----F---------DCVFTAQETRDAERECKKHCEGNN---EYVTRYCGGLCSSNTGPQTFYCYLGCSHNAS-
55	A0A0U5KIV9_MEG-27	53.0%	3.5%	-----------MN-----LIQTLL-------W---------MIFMMIMNLTNEIKW--
56	A0A5K4F014_MEG-27	45.5%	4.9%	-----------MN-----LIQTLL ------W---------MIFMMIMNLTNEIKWV---
57	G4LYD1_MEG-5	53.0%	2.5%	-----------MRRNY-LLLYICIIV-----F---------ILLKEINASGRQPKFVNVDTDGNL-------R-SGG--SS-------------------
58	G4VTX1_MEG-6	65.2%	1.5%	-----------MV----------------------------------QNPKN----TKK----IN-----------------------------------
59	A0A3Q0KTG4_MEG-11	71.2%	4.1%	-----------MK--LTHILLICFISFL---F-------------FTYVQCDGEEEENE---
60	A0A0U5KLL2_MEG-29	69.7%	4.5%	---------MLNK-----LLLQLFILV-----------T---IIIIHDVKCGGEEET---
61	A0A0U5KEW2_MEG-32.1	75.8%	1.1%	--------MYYRH--YLLAIINVIVLS-----------T-------MIQ-YVIGGSIFGDDT--------------------------------------
62	A0A0U5KJ28_MEG-32.2	78.8%	3.9%	MKETTVMHHYHHP--HPNHRLLTVISA---------------IVLLTIVHDVKGSGLFDDDITT------------------------------------
63	A0A5K4EK08_MEG-14	100.0%	5.3%	YLLCKKEYSIMNR--FFWTVTQCTILL-----------V---IICNLNTMKATSANSRTHGATSTS---THGATSTAKPAASTPPKAAATSTIKPTVTTP
64	A0A1C9A1H6_MEG-14	100.0%	5.0%	----------MNR--FFWTVTQCTILL-----------V---IICNLNTMKATSANSRTHGATSTS---THGATSTAKPAASTP---------------P
65	Q8ITD5_MEG-14	100.0%	4.5%	----------MNR--FFWTVTQCTILL-----------V---IICNLNTMKATSANSRTHGATSTS---THGATSTAKPAASTPPKAAATSTIKPTVTTP
66	A0A1C9A1I1_MEG-14	100.0%	5.7%	----------MNR--FFWTVTQCTILL-----------V---IICNLNTMKATSANSRTHGATSTS---THGATSTAKPAASTPPKAAATSTIKPTVTTP
67	A0A1C9A1I0_MEG-14	100.0%	5.8%	----------MNR--FFWTVTQCTILL-----------V---IICNLNTMKATSANSRTHGATST----THGATSTAKPAASTP---------------P
68	A0A1C9A1I5_MEG-14	100.0%	5.1%	----------MNR--FFWTVTQCTILL-----------V---IICNLNTMKATSANSRTHGATST----THGATSTAKPAASTP---------------P
69	A0A1C9A1J0_MEG-14	100.0%	4.9%	----------MNR--FFWTVTQRTILL-----------V---IICNLNTMKATSANSRTHGATSTR---THGATSTAKPAASTP--------IKPTVTTP
70	A0A1C9A1I3_MEG-14	100.0%	4.6%	----------MNR--FFWTVIQCTILL-----------V---IICNLNTMKATSANSRTHGATSTR---THGATSTAKPAASTPPKAAATSTIKPTVTTP
71	A0A1C9A1I4_MEG-14	100.0%	5.2%	----------MNR--FFWTVTQCTILL-----------V---IICNLNTMKATSANSRTHGATSTR---THGATSTAKPAASTPPKAAATSTIKPTVTTP
72	C4QPR8_MEG-2	69.7%	2.7%	-----------MH--GIWCKVPVSVVI------------------------------WIHSTLFQF---TFKVFYELKQNNTFPLP--------------
73	A0A3Q0KTV3_MEG-2	81.8%	2.8%	-----------MH--GIWCKVPVSVVI------------------------------WIHSTLFQF---TFKVFYELKQNNTFPLP--------------
74	C4QPR9_MEG-2	62.1%	2.7%	---------------QKAISQRPFAVI---
75	A0A0U5KJN7_MEG-10.2	48.5%	10.6%	----------MISLLLFGLLLL----Q----------------SCLYCSSDN----ENAGTTTEKPTSF---WKRFFDFFN-------------------
76	A0A0U5KFM1_MEG-31	42.4%	2.1%	-----------MH-----CVLLLLSL-------------F--AVCSVIMPTVKSGGSASGGSTEVD---LMHKRGKDR----------------------
77	C4QPS0_MEG-2	62.1%	3.8%	----------MTA--KGSVAMASFVLV------------Y--
78	C4QG05_MEG-2	74.2%	5.2%	----------M-----------CLTIF------------Y-VIHLLAI-FS--DSTEWVITCN-------------------------------------
79	D7PD69_MEG-2.4	75.8%	3.6%	----------M-----------CLTIF------------Y-VIHLLAI-FS--DSTEWVI--
80	A0A3Q0KR24_MEG-2	57.6%	8.7%	----------M-----------CLTIF------------Y-VIHLLAI-FS--DSNEWVITCN-------------------------------------
81	C4QPR6_MEG-2	75.8%	1.1%	----------MER--FKSSYF--YFEI------------Y---LLC--FT--ETVCCESEDGKAG-------------------------------
82	A0A5K4FFX0_MEG-2.2	62.1%	0.8%	MYCQSFTLLNRDYISNVTKQSKHRLYNTMKLSGANCLVVFSLLQLLVA-FSHC-----KLMSHNMQQDS
83	A0A5K4FDB9_Uncharacterized	74.2%	3.0%	----------------------------MKLSGANCLVVFSLLQLLVA-FSHCDIS-----DI--------------------------------------
84	D7PD77_MEG-2.2	74.2%	2.0%	----------------------------MKLSGATCLVVFSLLQLLVA-FSHCDIS-----AI--------------------------------------
85	D7DP78_MEG-2.1	92.4%	8.6%	----------------------------MKLSGANCLVVFSLLQLLVA-FSHCDIND-----ITCNKTV-------------------------------
86	D7DP76_MEG-2.1	53.0%	4.8%	----------------------------MKLSGANCLVVFSLLQLLVA-FSHCDIND---
87	D7DP75_MEG-2.1	31.8%	6.8%	----------------------------MKLSGANCLVVFSLLQLLVA-LSH--
	consensus/100%			....
	consensus/90%			........................h...
	consensus/80%			...............h.hhhh.h.lh..................h.......tt.t.h..
	consensus/70%			...............h.hhhh.shlhh....h............h.h.....psttph.ptht.....................................

```
                                     cov    pid 101        .         .         .         .         :         .         .         .         .         200
 1 A0A0U5KKP6_MEG-28             100.0% 100.0%    --------------------------------------------------IITTIEI--QNIRSAFKKRPPASFVILENMTSTDRFRKLLYHCFT---SF
 2 A0A0U5FZ31_MEG-26              51.5%   1.9%    ---------------------------------------------------TFKCLLSPLPESIGGKNK-------------------------------
 3 C4QKE8_MEG-4                   71.2%   2.5%    ----------------------YDDQLPDFPHKQLEE--------------EQNPFHKLSEVLNSGSV----------------VPLWLVNPIYYVLELF
 4 A0A5K4F4B1_MEG-4              100.0%   5.6%    KNVDIRI---------------IENKKDKHPTQ---------KTSDTHTIKRTTPKPTTPKQINDGTSDKPKSIADFFLINKPKVPLWIVNPLYYMVEKF
 5 A0A5K4F2K5_MEG-4               98.5%   4.4%    SDTHT-----------------IKRTTPKPTTPKQINDGTSDKTSDTHTIKRTTPKPTTPKQINDGTSDKPKSIADIFLINKPKVPLWIVNPLYYMVEKF
 6 A0A5K4F627_MEG-4               98.5%   4.6%    TQK----------------------TTPKPTTPKQINDGTSDKTSDTHTIKRTTPKPTTPKQINDGTSDKPKSIADIFLINKPKVPLWIVNPLYYMVEKF
 7 Q86D79_MEG-4                  100.0%   4.0%    PKPTTPKQINDGTSDKTSDTHTIKRTTPKPTTPKQINDGTSDKTSDTHTIKRTTPKPTTPKQINDGTSDKPKSIADIFLINKPKVPLWIVNPLYYMVEKF
 8 A0A3Q0KQX7_MEG-16              98.5%   1.7%    --------------------------------------------DFLTSLFDWNGDGYRGSGFNFYDFL---SLFFGLNKKDNRNNRRYRSGGGGGNGGL
 9 A0A5K4EU45_MEG-16              92.4%   2.9%    --------------------------------------------DFLTSLFDWNGDGYRGSGFNFYDFL---SLFFGLNKKDNRNNRRYRSGGGGGNGGL
10 A0A3Q0KLA7_MEG-13              93.9%   4.5%    ----------------------FNNTTVTSTTTEFNN---------TTVTSTTTEFTNKPKVENSTTDG---TTYTTTPSHFSTSTSTNDATNSKFQRIF
11 A0A5K4EL02_MEG-13             100.0%   5.6%    ----------------------FNNTTVTSTTTEFNN---------TTVTSTTTEFTNKPKVENSTTDG---TTYTTTPSHFSTSTSTNDATNSKFQRIF
12 A0A3Q0KQ39_MEG-10              42.4%   6.9%    ---------------------------------------------------TFCTFITFSNTIQNFIYG-------------------------------
13 G4LYD0_MEG-10                  40.9%   6.9%    ---------------------------------------------------TFCTFITFSNTIQNFIYG-------------------------------
14 G4VCW5_MEG-8                   98.5%   2.6%    CKLP------------------NPSQKGFTNTTSLPH-------TQSHNSTVAPSVPKPTRQEIPRSGTIVNGTKPTPGKPVVNGTKPTPGKPESFLKRV
15 A0A3Q0KKW2_MEG-9               69.7%   5.6%    ---------------------------------------------------HESSTEGQNHEELAAAAG---AHFLQFLNGC--------------FLNM
16 G4VLP3_MEG-8                  100.0%   5.0%    GTNN------------------KDTKSPNPNT--------------------TEAKSLSLKERIMNKFN---SIFGEEEYNPPKDSDFTERLWMLFKHCF
17 G4V7W5_MEG-7                  100.0%   5.2%    ----------------------STDKIPVNK--------------------TMKIQTTPSKEKKQKPDP---KRYKRSSYQKDKKAKSSSSTLTIGYPIL
18 A0A5K4EUJ7_MEG-7-iso2          98.5%   2.7%    ----------------------STDKIPVNK--------------------TMKIQTTPSKEKKQKPDP---KRYKRSSYQKDKKAKSSSSTLTIGYPIL
19 A0A5K4EKN1_MEG-1               95.5%   4.5%    FREI------------------NIDKDYLCTYPT----------NFSHGLITYCTKSNDERDLLSYEED---HIALYVIQPT-NHCQRYEGSS--IKKRL
20 D7PD99_MEG-1                   95.5%   4.9%    FREI------------------------------------------SHGLITYCTKSNDERDLLSYEED---HIALYVIQPT-NHCQRYEGSS--IKKRL
21 D7PD79_MEG-1                   95.5%   4.5%    FREI------------------NIDKDYLCTYPT----------NFSHGLITYCTKSNDERDLLSYEED---HIALYVIQPT-NHCQRYEGSS--IKKRL
22 D7PD91_MEG-1                   95.5%   5.0%    FREI------------------NIDKDYLCTYPT----------NFSHGLITYCTKSNDERDLLSYEED---HIALYVIQPT-NHCQRYEGSS--IKKRL
23 D7PD95_MEG-1                   95.5%   5.2%    FREI------------------NIDKDYLCTYPT----------NFSHGLITYCTKSNDERDLLSYEED---HIALYVIQPT-NHCQRYEGSS--IKKRL
24 D7PD89_MEG-1                   98.5%   4.9%    FREI------------------------------------------SHGLITYCTKSNDERDLLSYEED---HIALYVIQPT-NHCQRYEGSSSSVSQKP
25 A0A3Q0KKC4_MEG-1               98.5%   4.5%    FREI------------------NIDKDYLCTYPT----------NFSHGLITYCTKSNDERDLLSYEED---HIALYVIQPT-NHCQRYEGSSSLVSQKP
26 D7PD88_MEG-1                   98.5%   4.7%    FREI-------------------------CTYPT----------NFSHGLITYCTKSNDERDLLSYEED---HIALYVIQPT-NHCQRYEGSSSLVSQKP
27 D7PD93_MEG-1                   98.5%   4.7%    FREI------------------NIDKDYLCTYPT----------NFSHGLITYCTKSNDERDLLSYEED---HIALYVIQPT-NHCQRYEGSSSLVSQKP
28 D7PD94_MEG-1                   98.5%   4.7%    FREI------------------NIDKDYLCTYPT----------NFSHGLITYCTKSNDERDLLSYEED---HIALYVIQPT-NHCQRYEGSSSLVSQKP
29 D7PD83_MEG-1                   98.5%   4.5%    FREI------------------NIDKDYLCTYPT----------NFSHGLITYCTKSNDERDLLSYEED---HIALYVIQPT-NHCQRYEGSSSLVSQKP
30 D7PD84_MEG-1                   98.5%   5.0%    FREI------------------NIDKDHLCTYPT----------NFSHGLITYCTKSNDERDLLSYEED---HIALYVIQPT-NHCQRYEGSSSLVSQKP
31 D7PD86_MEG-1                   98.5%   5.6%    FREI------------------------------------------SHGLITYCTKSNDERDLLSYEED---HIALYVIQPT-NHCQRYEGSSSLVSQKP
32 A0A5K4FAB4_Uncharacterized     95.5%   2.9%    IREIIAANYSPIIHNEEKKNISNREKDYLCTCPT----------YFNHGVITYCTKSNYEKDSLQYEDD---FMDLFVKKHNKDDCQHYGGYSSLEYRNP
33 A0A5K4F8B3_MEG-1              100.0%   3.2%    IREIIAANYSPIIHNEEKKNISNREKDYLCTCPT----------YFNHGVITYCTKSNYEKDSLQYEDD---FMDLFVKKHNKDDCQHYGGYSSLEYRNP
34 A0A5K4F8U8_MEG-1              100.0%   3.4%    IREIIAANYSPIIHNEEKKNISNREKDYLCTCPT----------YFNHGVITYCTKSNYEKDSLQYEDD---FMDLFVKKHNKDDCQHYGGYSSLEYRNP
35 A0A0U5KI45_MEG-30              59.1%   8.6%    ---------------------------------------------------FFTTTPVPNKGLLDKLLD---GLYQFFNRH-------------------
36 A0A5K4E9G8_MEG-15              95.5%   2.8%    --------------------------------------------TTATASHTDKTVQKKCLNKMTPQDL---ISLLFSLIPQIKTIEFSQNENLLKLATI
37 A0A5K4E9M7_MEG-15              93.9%   1.8%    TTLS------------------HHNTVPAKTTRKSQH-------PNTTPSHTDKTVQKKCLNKMTPQDL---ISLLFSLIPQIKTIEFSQNENLLKLATI
38 A0A3Q0KC91_MEG-15              95.5%   2.8%    ------------------------------------H-------PNTTPSHTDKTVQKKCLNKMTPQDL---ISLLFSLIPQIKTIEFSQNENLLKLATI
39 G4VMN2_MEG-15                  95.5%   2.3%    TTLS------------------HHNTVPAKTTRKSQH-------PNTTPSHTDKTVQKKCLNKMTPQDL---ISLLFSLIPQIKTIEFSQNENLLKLATI
40 A0Q3Q0KQ41_MEG-12              39.4%   2.4%    -------------------------------------------VFCSVSNMVN----------------WIFG-----------------------------
41 A0A5K4EPC8_MEG-3.2            100.0%   1.9%    NQND------------------FDKCLPKCNGSPQL---------------TESSCQNDCGRPHTLNCV---VSFVVEMLETHFHCVCITAIREMVRETL
42 D7PD63_MEG-3.3                100.0%   4.8%    KQSE------------------FDNCKTKCDGGVQLT-----KEACLSNCGLITTHPELCDAVCGGNDG---GSFPICLYNCDQEHTDPRKDGADGSEDF
43 D7PD64_MEG-3.3                100.0%   4.9%    KQSE------------------FDNCKTKCDGGVQLT-----KEACLSNCGLITTHPELCDAVCGGNDG---GSFPICLYNCDQEHTDPRKDGADGSEDF
44 D7PD62_MEG-3.3                100.0%   4.6%    KQSE------------------FDNCKTKCDGGVQLT-----KEACLSNCGLITTHPELCDAVCGGNDG---GSFPICLYNCDQKHTDPRKDGADGSEDF
45 A0A3Q0KMS0_MEG-3              100.0%   4.6%    KQSE------------------FDNCKTKCDGGVQLT-----KEACLSNCGLITTHPELCDAVCGGNDG---GSFPICLYNCDQEHTDPRKDGADGSEDF
46 D7PD57_MEG-3.2                 86.4%   3.4%    NQND------------------FDKCLPKCNG--------------QNDCGRVTTHPELCGIVCGGNDG---GSFPICLYNCDQGN---------GSGNF
47 D7PD60_MEG-3.2                 86.4%   3.4%    NQND------------------FDKCLPKCNG--------------QNDCGRVTTHPELCGIVCGGNDG---GSFPICLYNCDQGN---------GSGNF
48 D7PD53_MEG-3.2                 86.4%   3.4%    NQND------------------FDKCLPKCNGSPQLT-----ESSCQNDCGRVTTHPELCGIVCGGNVG---DSFPLCLYNCDQGN---------GSGNF
49 D7PD52_MEG-3.2                 86.4%   3.2%    NQND------------------FDKCLPKCNGSPQLT-----ESSCQNDCGRVTTHPELCGIVCGGNVG---DSFPLCLYNCDQGN---------GSGNF
50 D7PD54_MEG-3.2                 86.4%   3.4%    NQND------------------FDKCLPKCNGSPQLT-----ESSCQNDCGRVTTHPELCGIVCGGNVG---DSFPLCLYNCDQGN---------GSGNF
51 D7PD51_MEG-3.1                 84.8%   1.9%    TQDD------------------FDKCLPKCNDRVQLT-----EENCRNDCGRVTSHHESCGDVCGGNHG---GSFPLCSYNCDQEHPR----------EY
52 A0A3Q0KMU6_MEG-3               90.9%   1.9%    TQDD------------------FDKCLPKCNDRVQLT-----EENCRDDCGRVTSHHELCGDVCGGNHG---GSFPLCLYNCDQEHPRE------YERGY
```

```
53 D7PD49_MEG-3.1                  90.9%   1.9%    TQDD------------------FDKCLPKCNDRVQLT-----EENCRDDCGRVTSHHELCGDVCGGNHG---GSFPLCLYNCDQEHPRE------YERGY
54 D7PD50_MEG-3.1                  68.2%   2.3%    TQDD------------------FDKCLPKCNDRVQLT-----EENCRNDCGRVTSHHELCGDVCGGNHG---GSFPLCFFQSSSSDK-------------
55 A0A0U5KIV9_MEG-27               53.0%   3.5%    -------------------------------------------VNCSHELNEHTSETSL--------RGWIHTVFSFLFHNF------------------
56 A0A5K4F014_MEG-27               45.5%   4.9%    --------------------------------------------NCSHELNEHTSETSL--------RRWIHTVFSFLFHNF------------------
57 G4LYD1_MEG-5                    53.0%   2.5%    --------------------------------------------DISDMFGQNKTLGTAFKTLLHNL--WDLLKQSLGLP--------------------
58 G4VTX1_MEG-6                    65.2%   1.5%    --------------RTIRR--ST-KTVIVITDRVQNI--------VLG---HRLLHHRIPTIKRSKSHG---INKNETVSNL------------------
59 A0A3Q0KTG4_MEG-11               71.2%   4.1%    ----------------------EEEKPPQPDVP--------------------HGKHPLLRKAFLTAPSW--LHMPFSIAGA------------------
60 A0A0U5KLL2_MEG-29               69.7%   4.5%    ----------------------TTTTLPT-TTS-------------------VAIKGTISAYTVMMGLS------------------------IYVI---
61 A0A0U5KEW2_MEG-32.1             75.8%   1.1%    --------------------------------------------STTKNMTTTTKASSANSLEVS----WLAISSIS----------------MIVIGLI
62 A0A0U5KJ28_MEG-32.2             78.8%   3.9%    R-----------------------------TTAP-----------------TTT-SGSVSSFQVS----WLALSSVF----------------MIVLGLI
63 A0A5K4EK08_MEG-14              100.0%   5.3%    KAAA------------------TSTIKP-----------------------TVTTKPSPAKPAASNTAK---PAASTPKKPHDERAVLAAAAVPIVLGVI
64 A0A1C9A1H6_MEG-14              100.0%   5.0%    KAAA------------------TSTIKPTVTTPKAAA-------TSTIKPTVTTSKPSPAKPAASNTAK---PAASTPKKPHDERAVLAAAAVPIVLGVI
65 Q8ITD5_MEG-14                  100.0%   4.5%    KAAA------------------TSTIKPTVTTPKAAA-------TSTIKPTVTTSKPSPAKPAASNTAK---PAASTPKKPHDERAVLAAAAVPIVLGVI
66 A0A1C9A1I1_MEG-14              100.0%   5.7%    KAAA------------------TSTIKP-----------------------TVTTKPSPAKPAASNTAK---PAASTPKKPHDERAVLAAAAVPIVLGVI
67 A0A1C9A1I0_MEG-14              100.0%   5.8%    KAAA------------------TSTIKPTVTTPKAAA--------TSTIKPTVTTKPSPAKPAASNTAK---PAASTPKKPHDERAVLAAAAVPIVLGVI
68 A0A1C9A1I5_MEG-14              100.0%   5.1%    KAAA------------------TSTIKPTVTTPKAAA-------TSTTEPTVTTSKPSPAKPAASNTAK---PAASTPKKPHDERAVLAAAAVPIVLGVI
69 A0A1C9A1J0_MEG-14              100.0%   4.9%    KAAA------------------TSTTEPTVTTPKAAA-------TSTTEPTVTTSKPSPAKPAASNTAK---PAASTPKKPHDERAVLAAAAVPIVLGVI
70 A0A1C9A1I3_MEG-14              100.0%   4.6%    KAAA------------------TSTTEPTVTTPKAAA-------TSTTEPTVTTSKPSPAKPAASNTAK---PAASTPKKPHDERAVLAAAAVPIVLGVI
71 A0A1C9A1I4_MEG-14              100.0%   5.2%    KAAA------------------TSTTEPTVTTPKAAA--------TSTTEPTVTTKPSPAKPAASNTAK---PAASTPKKPHDERAVLAAAAVPIVLGVI
72 C4QPR8_MEG-2                    69.7%   2.7%    ----------------------GDGWTITCNE-------------------TYCCENTDNGKLCCDGEY---CSASISKLDP------------------
73 A0A3Q0KTV3_MEG-2                81.8%   2.8%    ----------------------GDGWTITCNE-------------------TYCCENTDNGKLCCDGEY---CSASISNHQDLTKHQQNL----------
74 C4QPR9_MEG-2                    62.1%   2.7%    --------------------------------------------------KMVVTVDNPELTLKNYLRK---------------------------AQMI
75 A0A0U5KJN7_MEG-10.2             48.5%  10.6%    ---------------------------------------------------FICTLNQTWSTIRNFF-----GIAL------------------------
76 A0A0U5KFM1_MEG-31               42.4%   2.1%    ---------------------------------------------------EDKRRKDYIKELVKNATG-------------------------------
77 C4QPS0_MEG-2                    62.1%   3.8%    ---------------------------------------------------------DPSVAVKNYRQQ------VLMATKIKEVCQKFRG---------
78 C4QG05_MEG-2                    74.2%   5.2%    --------------------------KTTCCDEDGNS--------------KICCVGNDCKDVIKPRSS---GADDLNLFLRKRG----------MAYKL
79 D7PD69_MEG-2.4                  75.8%   3.6%    ----------------------TCNKTTCCD---------------EDGNSKICCVGNDCKDVIKPRSS---GADDLNLFLRKRGMAYKLGE---ILKKL
80 A0A3Q0KR24_MEG-2                57.6%   8.7%    --------------------------KTTCCDEDKNS--------------KICCVGNDCKDVIKPRSS---GADDFDLLKKLNSP--------------
81 C4QPR6_MEG-2                    75.8%   1.1%    ---------------------------------------------------SLCCEKNGCSVPSGTHDL---LSENYRRHQR-------------MKNYL
82 A0A5K4FFX0_MEG-2.2              62.1%   0.8%    ----------------------------------------------LLRQKTVKKVRTEERWLSNTPDL---LLGNYQRHQR-------------MKNYL
83 A0A5K4FDB9_Uncharacterized      74.2%   3.0%    ----------------------TCNKTVCCASEDGKT-------GS------LCCEKDGC--PS-TPDL---FLENYRRHRR-------------MKNYL
84 D7PD77_MEG-2.2                  74.2%   2.0%    ----------------------TCNKTVCCASEDGKT-------GS------LCCEKDGC--PS-TPDL---FLENYRRHRR-------------MKNYL
85 D7DP78_MEG-2.1                  92.4%   8.6%    ----------------------------CCASEDGKK-------GS------LCCEKDGCPIPS-TPDL---LLGNYQRHQR-------------MKNYL
86 D7DP76_MEG-2.1                  53.0%   4.8%    ---------------------ITCNKTVCCASEDGKI---------------------------------------------------------------
87 D7DP75_MEG-2.1                  31.8%   6.8%    ----------------------------------------------------------------------------------------------------
   consensus/100%                                  ....................................................................................................
   consensus/90%                                   .....................................................p...t..........................................
   consensus/80%                                   ...................................................hhssh.p.tt.h.t.t.........h.......................
   consensus/70%                                   ...................................................phsspsp.pp.hhs.ptt.....h.h.h..p.p...........h.t..
```

```
                              cov    pid 201         .         .         .         .    ] 246
 1 A0A0U5KKP6_MEG-28          100.0% 100.0%     S------------------TWMVLLG--------------------
 2 A0A0U5FZ31_MEG-26           51.5%   1.9%     ----------------------SCYP--------------------
 3 C4QKE8_MEG-4                71.2%   2.5%     P----------R-------AISYYFN--------------------
 4 A0A5K4F4B1_MEG-4           100.0%   5.6%     V----------Q-------IMGYLLEDDDTLELNLPKYYYDKSI--
 5 A0A5K4F2K5_MEG-4            98.5%   4.4%     V----------Q-------IMGYLLEDDDTLELNLPKYYYDKSI--
 6 A0A5K4F627_MEG-4            98.5%   4.6%     V----------Q-------IMGYLLEDDDTLELNLPKYYYDKSI--
 7 Q86D79_MEG-4               100.0%   4.0%     V----------Q-------IMGYLLEDDDTLELNLPKYYYDKSI--
 8 A0A3Q0KQX7_MEG-16           98.5%   1.7%     -------------------IRLFFAR--------------------
 9 A0A5K4EU45_MEG-16           92.4%   2.9%     -------------------IRLFFAR--------------------
10 A0A3Q0KLA7_MEG-13           93.9%   4.5%     Y----------M-------IVGLISLMAIN----------------
11 A0A5K4EL02_MEG-13          100.0%   5.6%     Y----------M-------IVGLISLMAIN----------------
12 A0A3Q0KQ39_MEG-10           42.4%   6.9%     ----------------------------------------------
13 G4LYD0_MEG-10               40.9%   6.9%     ----------------------------------------------
14 G4VCW5_MEG-8                98.5%   2.6%     GDGFFDLFSEQEFHPINHKSYLFNFWYLFRTSFLNLKNMKNLLLGS
15 A0A3Q0KKW2_MEG-9            69.7%   5.6%     D----------N-------LKKLVFPG-------------------
16 G4VLP3_MEG-8               100.0%   5.0%     L----------N-----FKNLAKIFST-------------------
17 G4V7W5_MEG-7               100.0%   5.2%     F----------I-------TTPFVISKFLL----------------
18 A0A5K4EUJ7_MEG-7-iso2       98.5%   2.7%     F----------I-------TTPFVISKFLL----------------
19 A0A5K4EKN1_MEG-1            95.5%   4.5%     L----------E-------SYLIITPI-------------------
20 D7PD99_MEG-1                95.5%   4.9%     L----------E-------SYLIITPI-------------------
21 D7PD79_MEG-1                95.5%   4.5%     L----------E-------SYLIITPI-------------------
22 D7PD91_MEG-1                95.5%   5.0%     L----------E-------SYLIITPI-------------------
23 D7PD95_MEG-1                95.5%   5.2%     L----------E-------SYLIITPI-------------------
24 D7PD89_MEG-1                98.5%   4.9%     E----------K-------ECPFCFD--------------------
25 A0A3Q0KKC4_MEG-1            98.5%   4.5%     E----------K-------ECPFCFD--------------------
26 D7PD88_MEG-1                98.5%   4.7%     E----------K-------ECPFCFD--------------------
27 D7PD93_MEG-1                98.5%   4.7%     E----------K-------ECPFCFD--------------------
28 D7PD94_MEG-1                98.5%   4.7%     E----------K-------ECPFCFD--------------------
29 D7PD83_MEG-1                98.5%   4.5%     E----------K-------ECPFCFD--------------------
30 D7PD84_MEG-1                98.5%   5.0%     E----------K-------ECPFCFD--------------------
31 D7PD86_MEG-1                98.5%   5.6%     E----------K-------ECPFCFD--------------------
32 A0A5K4FAB4_Uncharacterized  95.5%   2.9%     E----------K-------EITQCSIWETLS---------------
33 A0A5K4F8B3_MEG-1           100.0%   3.2%     E----------KVCPFCYEEITQCSIWETLS---------------
34 A0A5K4F8U8_MEG-1           100.0%   3.4%     E----------K-------EITQCSIWETLS---------------
35 A0A0U5KI45_MEG-30           59.1%   8.6%     ----------------------------------------------
36 A0A5K4E9G8_MEG-15           95.5%   2.8%     L----------E----------KIFEQQSRVEHSSPTKTPANKIFH
37 A0A5K4E9M7_MEG-15           93.9%   1.8%     L----------E----------KIFEQQSRVEHSSPTKTPANKIFH
38 A0A3Q0KC91_MEG-15           95.5%   2.8%     L----------E----------KIFEQQSRVEHSSPTKTPANKIFH
39 G4VMN2_MEG-15               95.5%   2.3%     L----------E----------KIFEQQSRVEHSSPTKTPANKIFH
40 A0Q3Q0KQ41_MEG-12           39.4%   2.4%     ----------------------------------------------
41 A0A5K4EPC8_MEG-3.2         100.0%   1.9%     T----------N-------VKQSATKWRDGEFP-------------
42 D7PD63_MEG-3.3             100.0%   4.8%     D----------K-------CKTKCYKMAGQ----------------
43 D7PD64_MEG-3.3             100.0%   4.9%     D----------K-------CKTKCYKMAGQ----------------
44 D7PD62_MEG-3.3             100.0%   4.6%     D----------K-------CKTKCYKMAGQ----------------
45 A0A3Q0KMS0_MEG-3           100.0%   4.6%     D----------K-------CKTKCYKMAGQ----------------
46 D7PD57_MEG-3.2              86.4%   3.4%     D----------E-------CKTKCYEMAGR----------------
47 D7PD60_MEG-3.2              86.4%   3.4%     D----------E-------CKTKCYEMAGR----------------
48 D7PD53_MEG-3.2              86.4%   3.4%     D----------E-------CKTKCYLMAGR----------------
49 D7PD52_MEG-3.2              86.4%   3.2%     D----------E-------CKTKCYEMAGR----------------
50 D7PD54_MEG-3.2              86.4%   3.4%     D----------E-------CKTKCYEMAGR----------------
51 D7PD51_MEG-3.1              84.8%   1.9%     E----------R-------GKTKRYAMEGR----------------
52 A0A3Q0KMU6_MEG-3            90.9%   1.9%     D----------K-------CKTKCYAMEGR----------------
```

```
53 D7PD49_MEG-3.1              90.9%   1.9%    D----------K-------CKTKCYAMEGR----------------
54 D7PD50_MEG-3.1              68.2%   2.3%    ----------------------------------------------
55 A0A0U5KIV9_MEG-27           53.0%   3.5%    ----------------------------------------------
56 A0A5K4F014_MEG-27           45.5%   4.9%    ----------------------------------------------
57 G4LYD1_MEG-5                53.0%   2.5%    ----------------------------------------------
58 G4VTX1_MEG-6                65.2%   1.5%    F----------P----------------------------------
59 A0A3Q0KTG4_MEG-11           71.2%   4.1%    -------------------VAAYVFYHFYG----------------
60 A0A0U5KLL2_MEG-29           69.7%   4.5%    -------------------HSFIVFKMM------------------
61 A0A0U5KEW2_MEG-32.1         75.8%   1.1%    N------------------GHLRRFIF-------------------
62 A0A0U5KJ28_MEG-32.2         78.8%   3.9%    N------------------SYTERSIF-------------------
63 A0A5K4EK08_MEG-14          100.0%   5.3%    G----------E-------VIGFILQYIAS----------------
64 A0A1C9A1H6_MEG-14          100.0%   5.0%    G----------E-------VIGFILQYIAS----------------
65 Q8ITD5_MEG-14              100.0%   4.5%    G----------E-------VIGFILQYIAS----------------
66 A0A1C9A1I1_MEG-14          100.0%   5.7%    G----------E-------VIGFILQYIAS----------------
67 A0A1C9A1I0_MEG-14          100.0%   5.8%    G----------E-------VIGFILQYIAS----------------
68 A0A1C9A1I5_MEG-14          100.0%   5.1%    G----------E-------VIGFILQ--------------------
69 A0A1C9A1J0_MEG-14          100.0%   4.9%    G----------E-------VIGFILQ--------------------
70 A0A1C9A1I3_MEG-14          100.0%   4.6%    G----------E-------VIGFILQ--------------------
71 A0A1C9A1I4_MEG-14          100.0%   5.2%    G----------E-------VIGFILQYIAS----------------
72 C4QPR8_MEG-2                69.7%   2.7%    -------------------PFSNCFQYVFVS---------------
73 A0A3Q0KTV3_MEG-2            81.8%   2.8%    -------------------LMSKKFKII------------------
74 C4QPR9_MEG-2                62.1%   2.7%    D----------K-------LREAVQKLGGR----------------
75 A0A0U5KJN7_MEG-10.2         48.5%  10.6%    ----------------------------------------------
76 A0A0U5KFM1_MEG-31           42.4%   2.1%    -----------T----------------------------------
77 C4QPS0_MEG-2                62.1%   3.8%    ----------------------------------------------
78 C4QG05_MEG-2                74.2%   5.2%    G----------E-------ILKKLN---------------------
79 D7PD69_MEG-2.4              75.8%   3.6%    N---------------------------------------------
80 A0A3Q0KR24_MEG-2            57.6%   8.7%    ----------------------------------------------
81 C4QPR6_MEG-2                75.8%   1.1%    K----------E------VCK---YFK-------------------
82 A0A5K4FFX0_MEG-2.2          62.1%   0.8%    E----------E------VQILHIYYI-------------------
83 A0A5K4FDB9_Uncharacterized  74.2%   3.0%    E----------E------VCK---YYI-------------------
84 D7PD77_MEG-2.2              74.2%   2.0%    E----------E------VCK---YYI-------------------
85 D7DP78_MEG-2.1              92.4%   8.6%    E----------E------VCENFIYTP-------------------
86 D7DP76_MEG-2.1              53.0%   4.8%    -------------------GENFIYTP-------------------
87 D7DP75_MEG-2.1              31.8%   6.8%    ------------------------YTP-------------------
   consensus/100%                              ..............................................
   consensus/90%                               ..............................................
   consensus/80%                               .......................h......................
   consensus/70%                               ....................h.hhh.....................
```
